# Supplementary material for: Ultrastretchable and superior healable supercapacitors based on a double cross-linked hydrogel electrolyte
Source: Nat Commun. 2019 Feb 1;10:536. doi: 10.1038/s41467-019-08320-z (PMC6358613; doi:10.1038/s41467-019-08320-z)
Supplement: Supplementary file 1 — Supplementary Information [file 41467_2019_8320_MOESM1_ESM.pdf]

Supplementary Information

Ultrastretchable and superior healable supercapacitors based on a double cross-linked hydrogel electrolyte

Li et al.

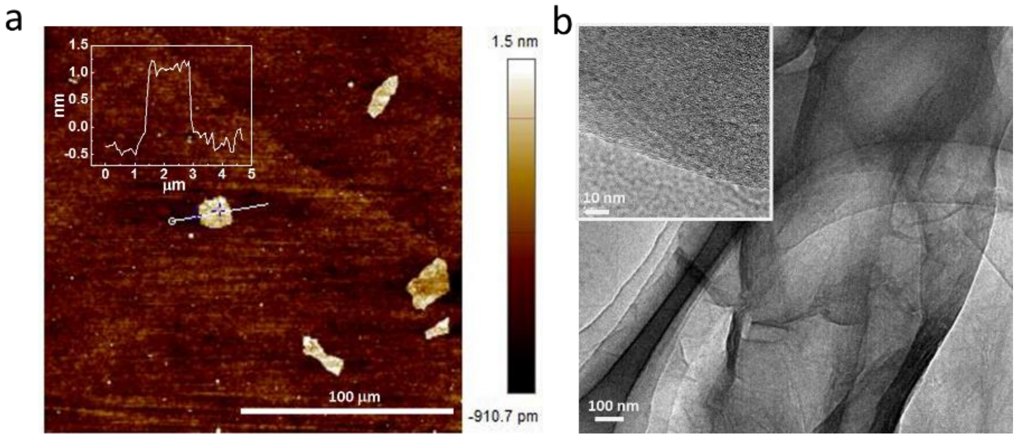

Supplementary Figure 1. Atomic force microscopy (**a**) and transmission electron microscope (**b**) images of the used graphene oxide sheets.

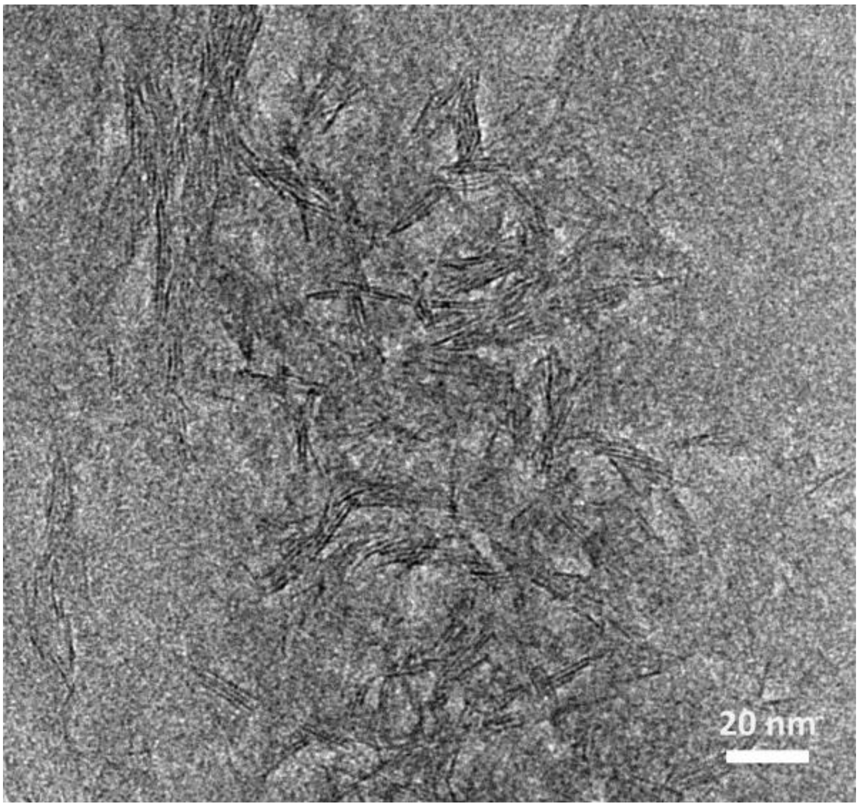

Supplementary Figure 2. TEM image of the used Laponite.

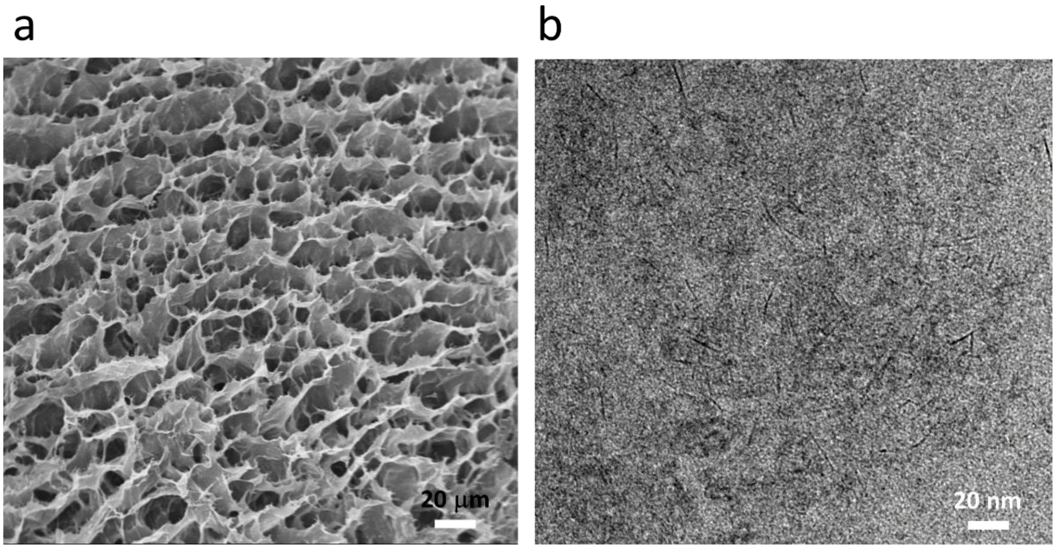

Supplementary Figure 3. (**a**) SEM of a freeze-dried nanocomposite hydrogel. (**b**) TEM images of nanocomposite hydrogel. The sample was prepared by grinding the dried hydrogel into fine power, and then dispersed in acetone for TEM characterization.

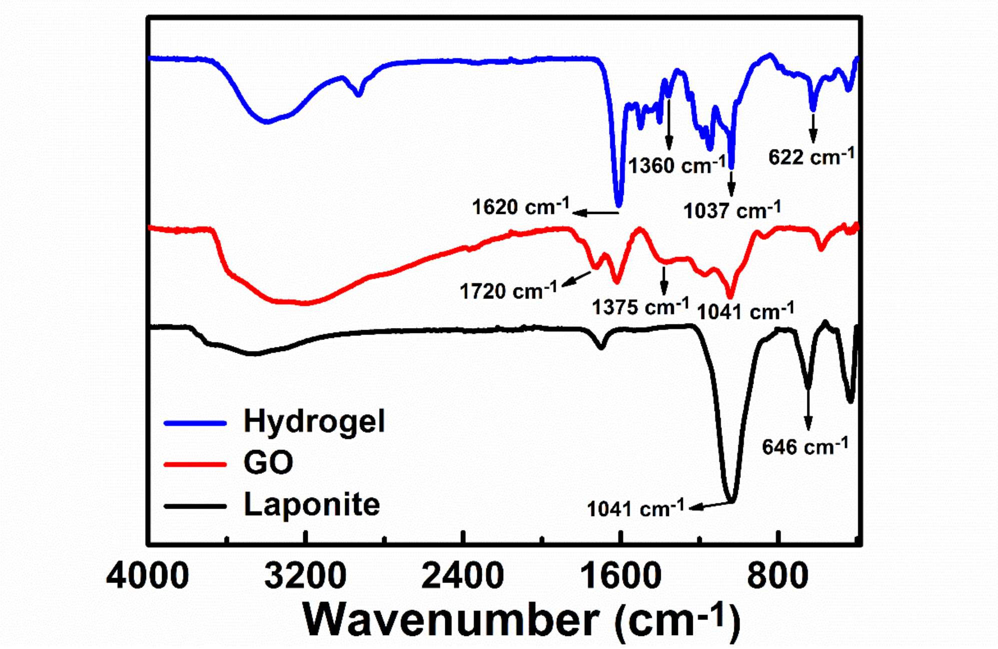

Supplementary Figure 4. FTIR spectra of Laponite, GO, and poly(AMPS-co-DMAAm)/Laponite/GO hydrogels.

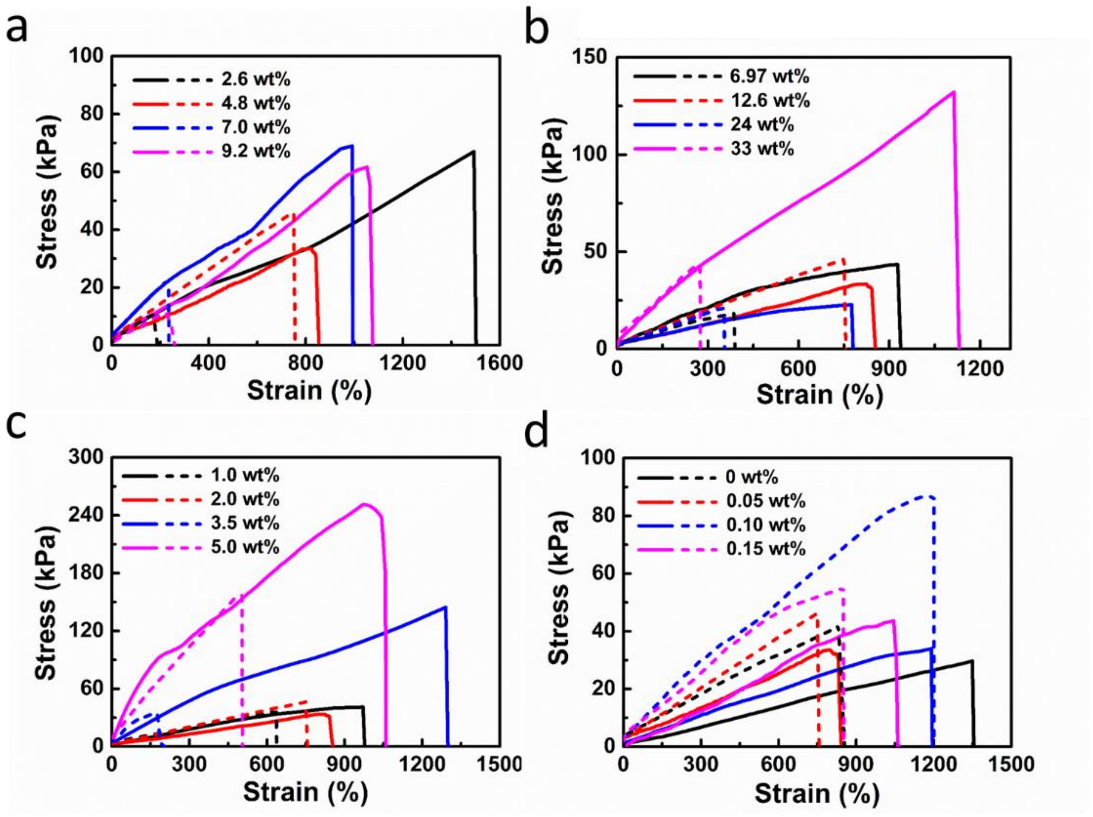

Supplementary Figure 5. Tensile stress-strain curves of hydrogels measured before (solid line) and after (dotted line) broken/healed with different contents of AMPS (**a**, The contents of DMAAm, Laponite and GO in precursor solution were fixed as 12.6 wt%, 2 wt% and 0.05 wt%, respectively.), DMAAm (**b**, The contents of AMPS, Laponite and GO in precursor solution were fixed as 4.8 wt%, 2 wt% and 0.05 wt%, respectively.), Laponite (**c**, The contents of AMPS, DMAAm and GO in precursor solution were fixed as 4.8 wt%, 12.6 wt% and 0.05 wt%, respectively.) and GO (**d**, The contents of AMPS, DMAAm and Laponite in precursor solution were fixed as 4.8 wt%, 12.6 wt% and 2 wt%, respectively.).

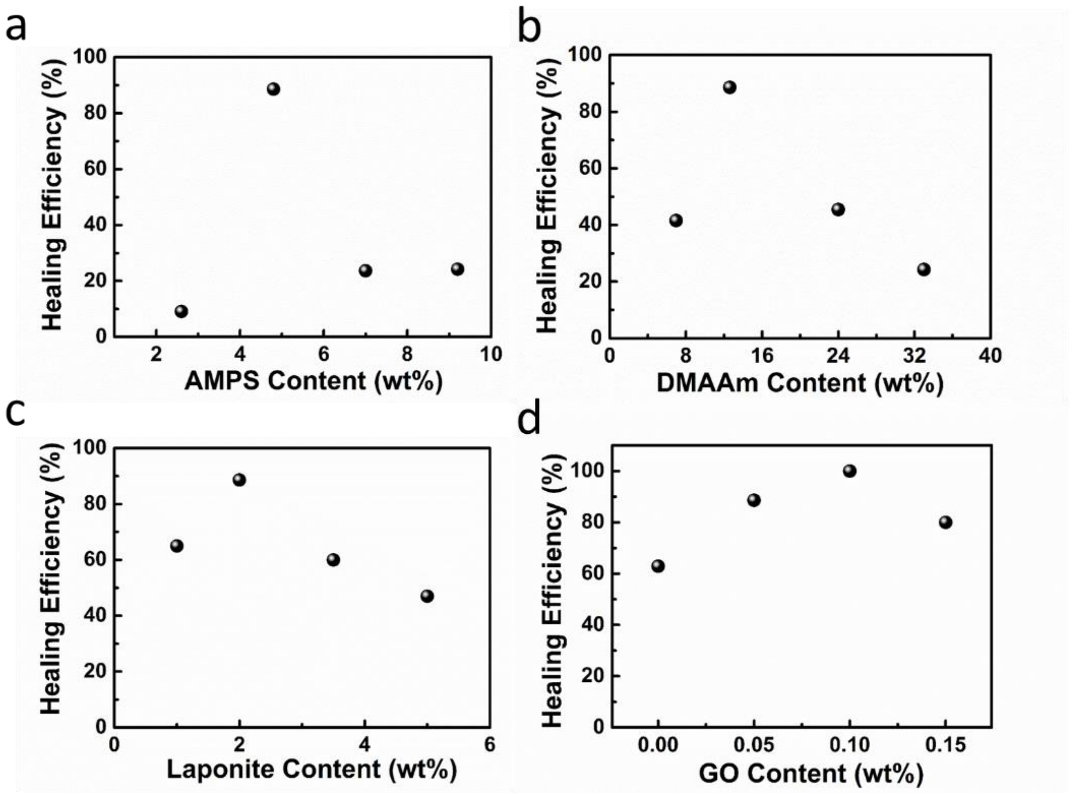

Supplementary Figure 6. Healing efficiencies of tensile strain of hydrogels with different contents of AMPS (**a**), DMAAm (**b**), Laponite (**c**) and GO (**d**), which were calculated based on the stress-strain curves in Supplementary Figure 5.

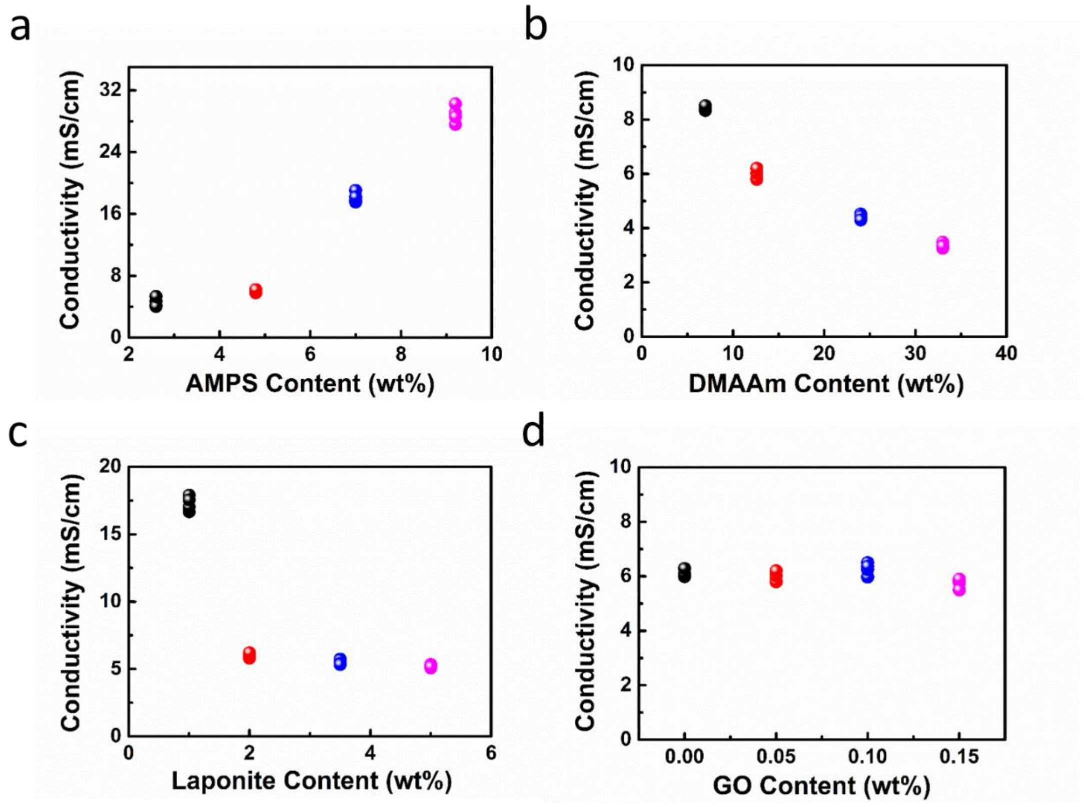

Supplementary Figure 7. Ionic conductivities of hydrogels with different contents of AMPS (**a**), DMAAm (**b**), Laponite (**c**) and GO (**d**). The detailed contents of all components were the same with Supplementary Figure 5. Ionic conductivities were calculated according to electrochemical impedance spectroscopies (EIS) measured by using electrochemical workstation.

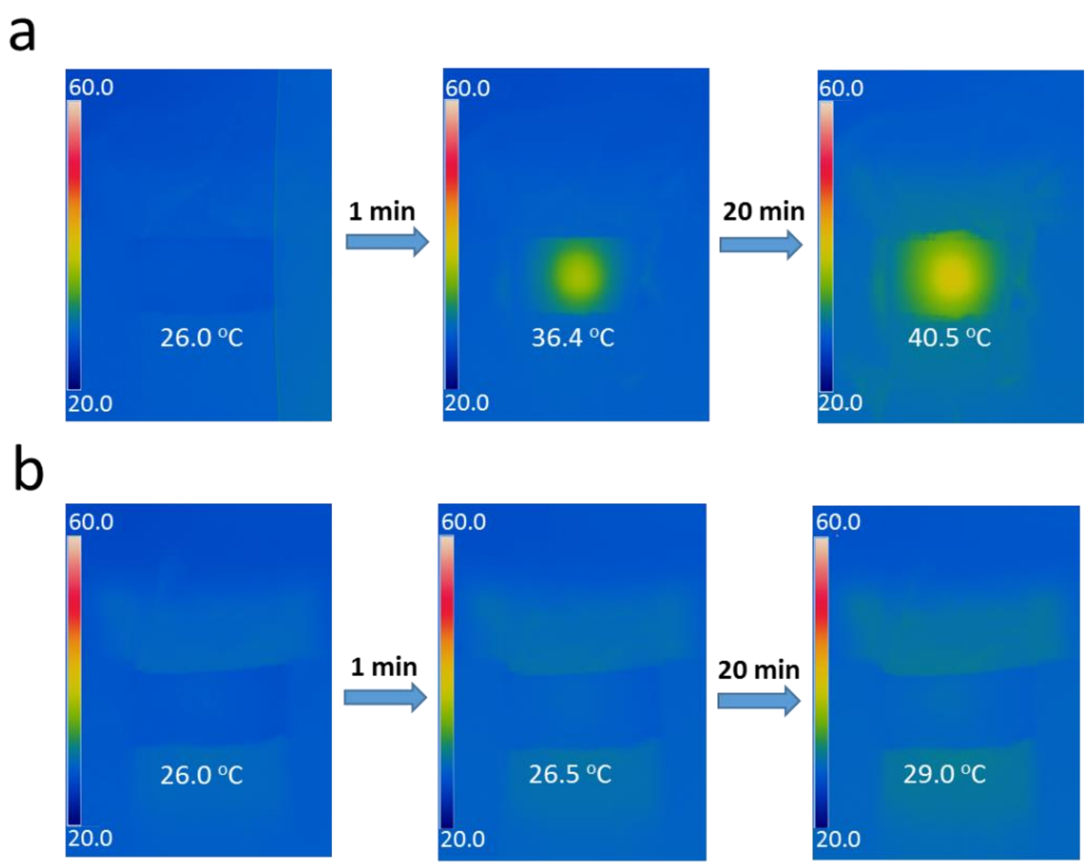

Supplementary Figure 8. Thermal mapping images of hydrogels with (a) and without (**b**) GO under persistent irradiation of infrared light.

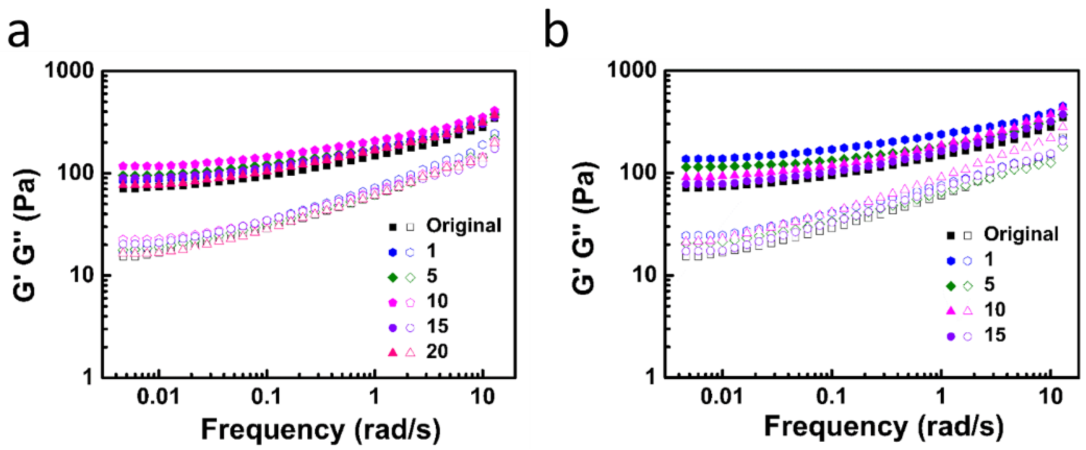

Supplementary Figure 9. Storage modulus G' (solid symbols) and loss modulus G'' (open symbols) for poly(AMPS-co-DMAAm)/Laponite/GO hydrogels during multiple broken/healed cycles under 808 nm infrared light irradiation for 20 min (**a**) and 80 ^{o}C for 60 min (**b**).

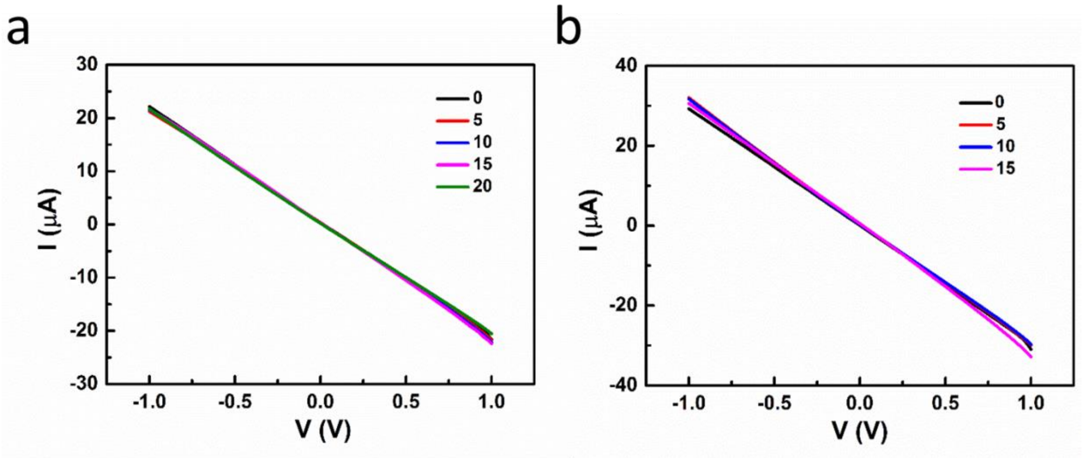

Supplementary Figure 10. Current-Voltage (I-V) curves of poly(AMPS-co-DMAAm)/Laponite/GO hydrogel films after cyclic broken/healed processes, measured by using a two-probe method. The broken hydrogel films were healed under irradiation of 808 nm infrared light for 20 min (**a**) or 80 ^{o}C for 60 min (**b**). The electrical resistances (R) of hydrogel films can be easily obtained from I-V curves. Electrical conductivities (σ) were calculated according to the equation of $\sigma = L/RS$, where R is the electrical resistance, L and S are the length and cross-sectional area of the hydrogel film for testing, respectively.

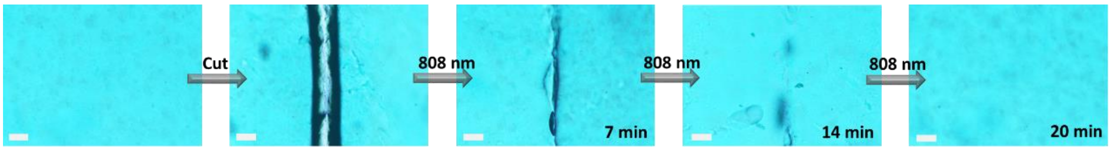

Supplementary Figure 11. Optical microscopic images of a poly(AMPS-co-DMAAm)/Laponite/GO hydrogel being cut and healed by 808 nm infrared light treatment for different time. The scale bar was 100 μm.

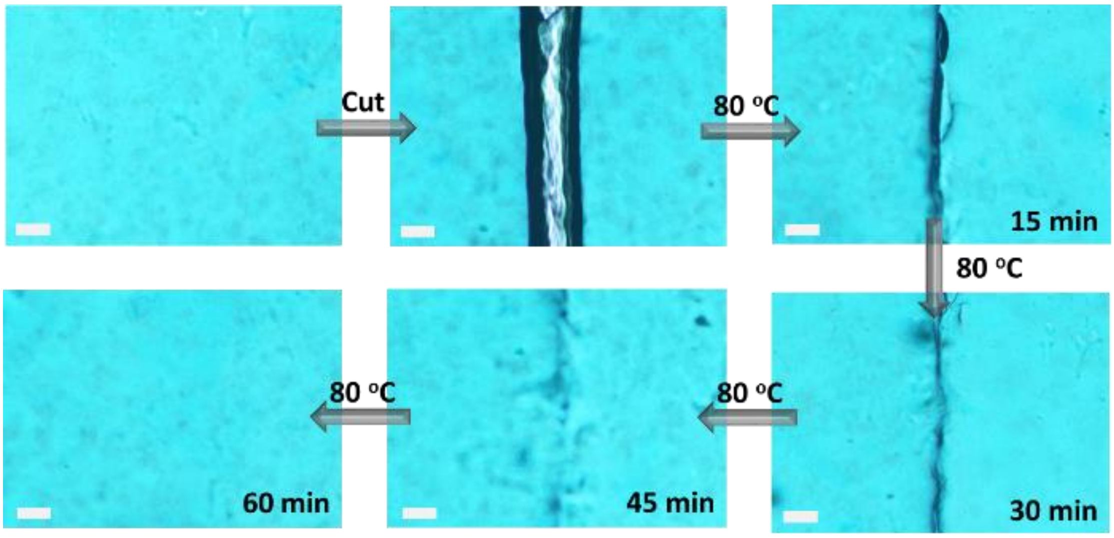

Supplementary Figure 12. Optical microscopic images of healing process for a poly(AMPS-co-DMAAm)/Laponite/GO hydrogel being cut and treated by 80 ^{o}C for different time. The scale bar was 100 μm.

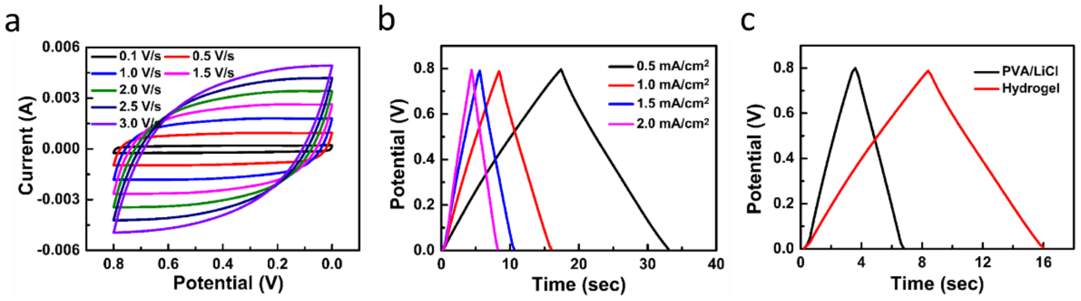

Supplementary Figure 13. (**a**) CV curves of an as-prepared supercapacitor based on poly(AMPS-co-DMAAm)/Laponite/GO hydrogel electrolyte at various scan rates from 0.1 to 3.0 V/s. (**b**) GCD curves of the supercapacitor at various charging/discharging currents from 0.025 to 5 mA. (**c**) GCD curves of the supercapacitor compared with device based on PVA/LiCl electrolyte.

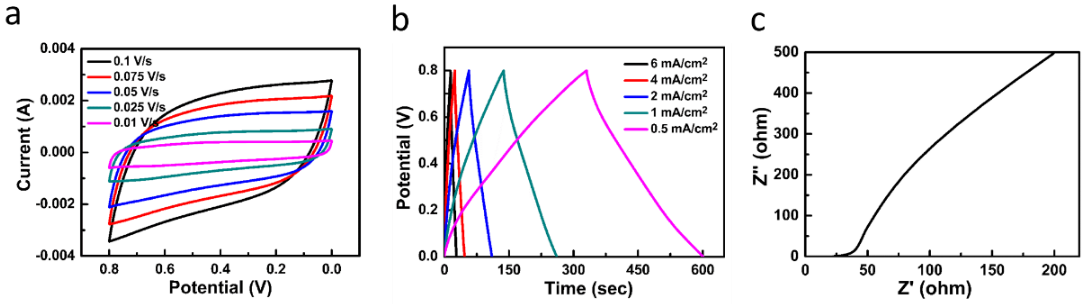

Supplementary Figure 14. CV (**a**), GCD (**b**) and EIS (**c**) curves of a supercapacitor by using CNT/PANI composite electrode and poly(AMPS-co-DMAAm)/Laponite/GO hydrogel electrolyte.

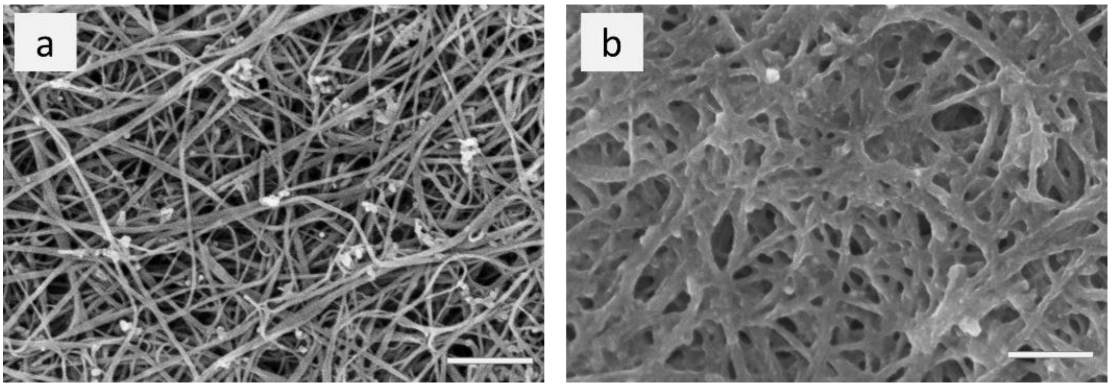

Supplementary Figure 15. SEM images of bare CNT film (**a**) and CNT/PANI composite film (**b**). The scale bar: 200 μm.

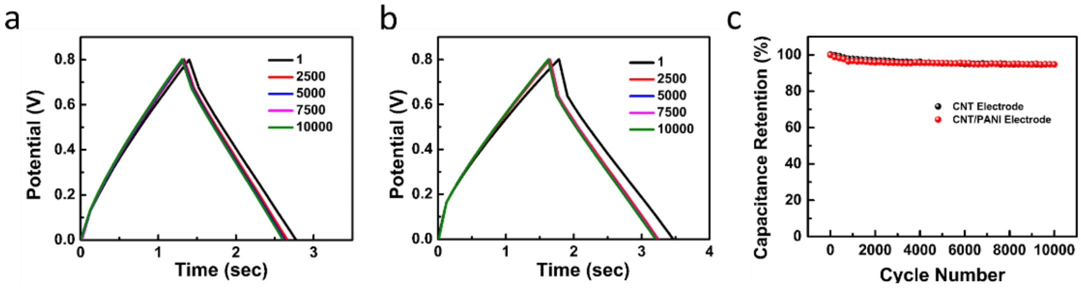

Supplementary Figure 16. GCD curves of supercapacitors based on hydrogel electrolyte with CNT films (1.2 mA/cm^2) (**a**) and CNT/PANI (49 mA/cm^2) composite (**b**) electrodes for 10000 charge/discharge cycles. (**c**) The specific capacitance retentions of the supercapacitors calculated from (**a**) and (**b**).

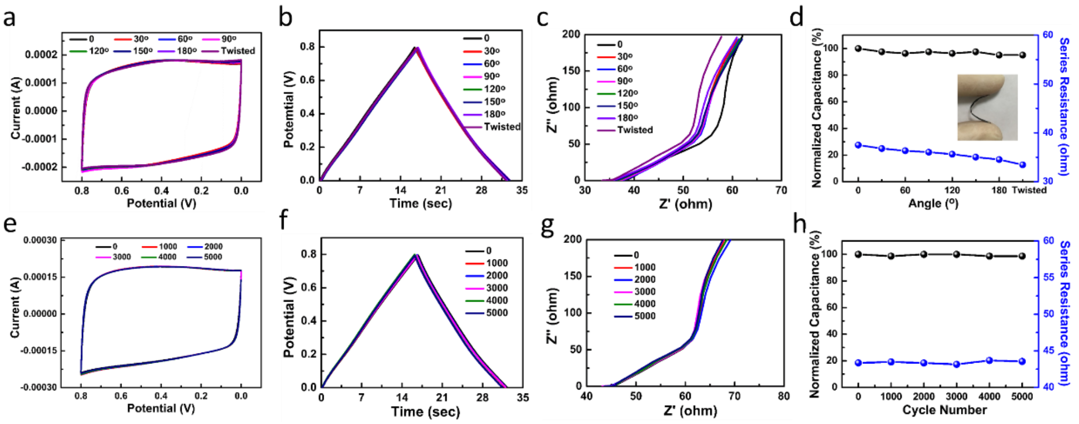

Supplementary Figure 17. CV (**a**), GCD (**b**), EIS (**c**) curves and specific capacitance retention (**d**) of hydrogels–based supercapacitors by using CNT film electrode under different bending angles. CV (**e**), GCD (**f**), EIS (**g**) curves and capacitance retention (**h**) of supercapacitor device by bending for different cycles.

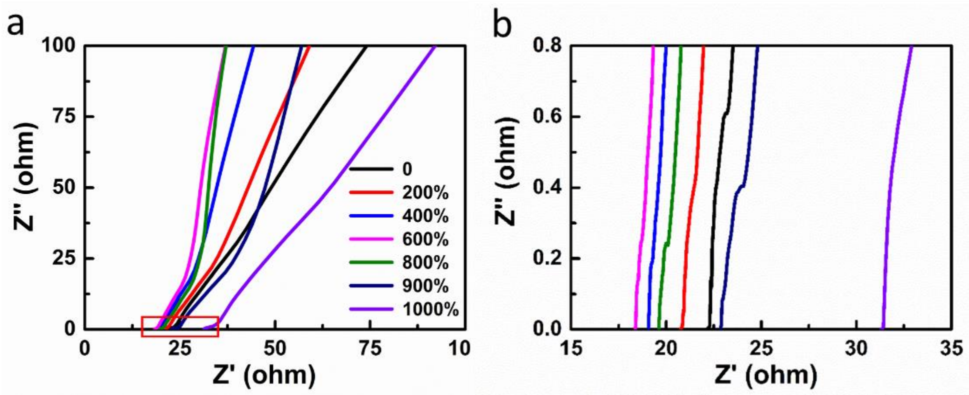

Supplementary Figure 18. EIS curves of a supercapacitor being stretched from 0% to 1000% strain (**a**). (**b**) The amplified red area in **a**.

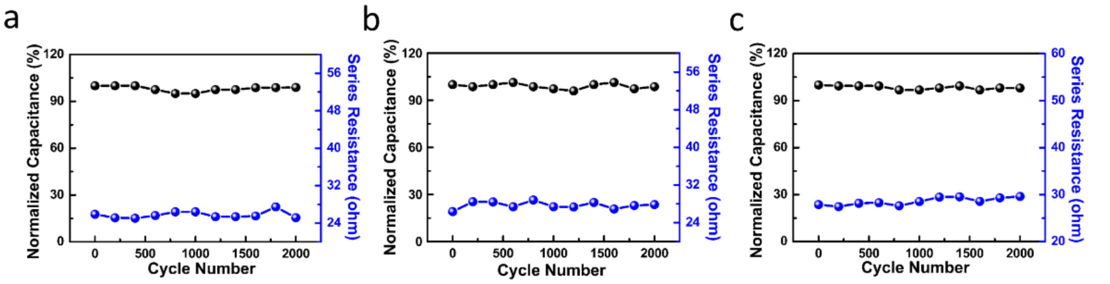

Supplementary Figure 19. Changes of specific capacitance and series resistance for supercapacitors based on the hydrogel electrolyte by stretched to strains of 100% (**a**), 200% (**b**) and 300% (**c**) for 2000 cycles.

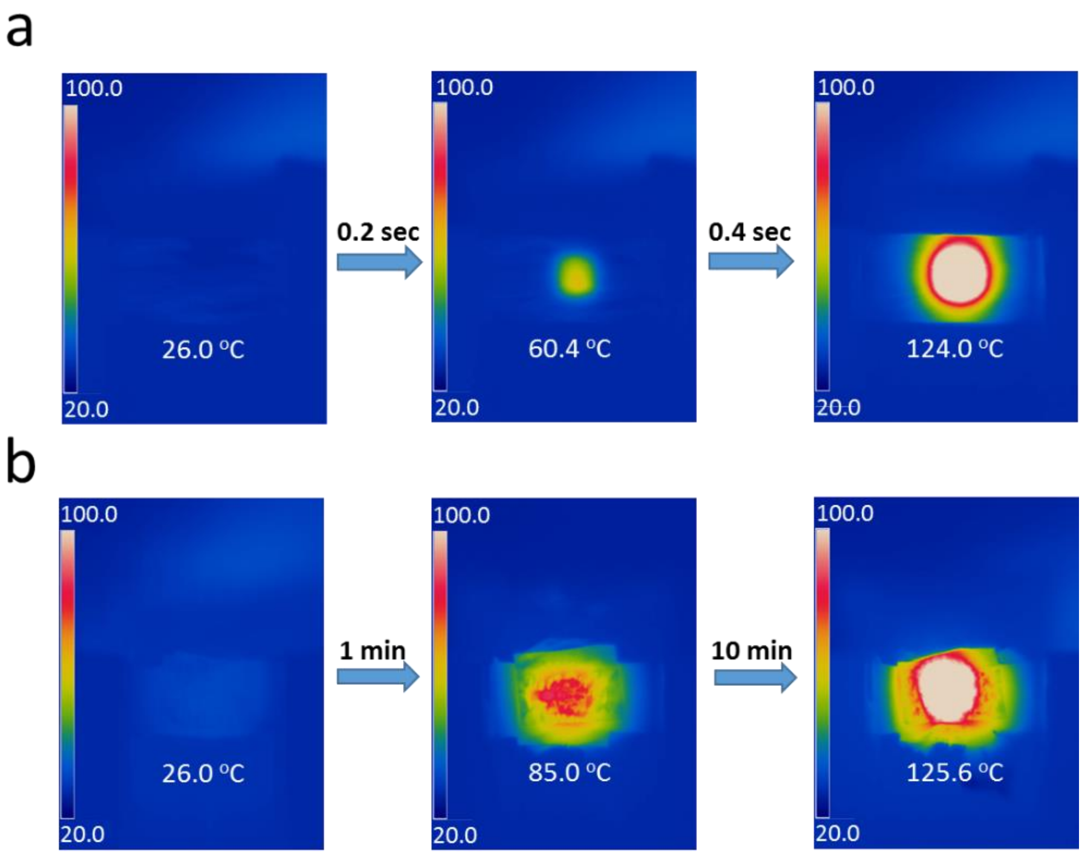

Supplementary Figure 20. Thermal mapping images of bare CNT film (**a**) and supercapacitor (**b**) with persistent irradiation of infrared light.

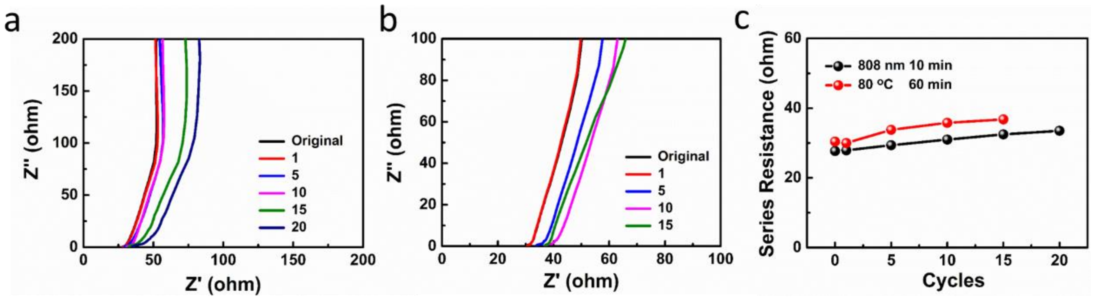

Supplementary Figure 21. EIS curves of supercapacitor devices repeatedly healed by irradiation with 808 nm infrared light for 10 min (**a**) and heating at 80 ^{o}C for 60 min (**b**) after different broken/healed cycles, respectively. (**c**) Series resistances of supercapacitors being broken/healed for different cycles.

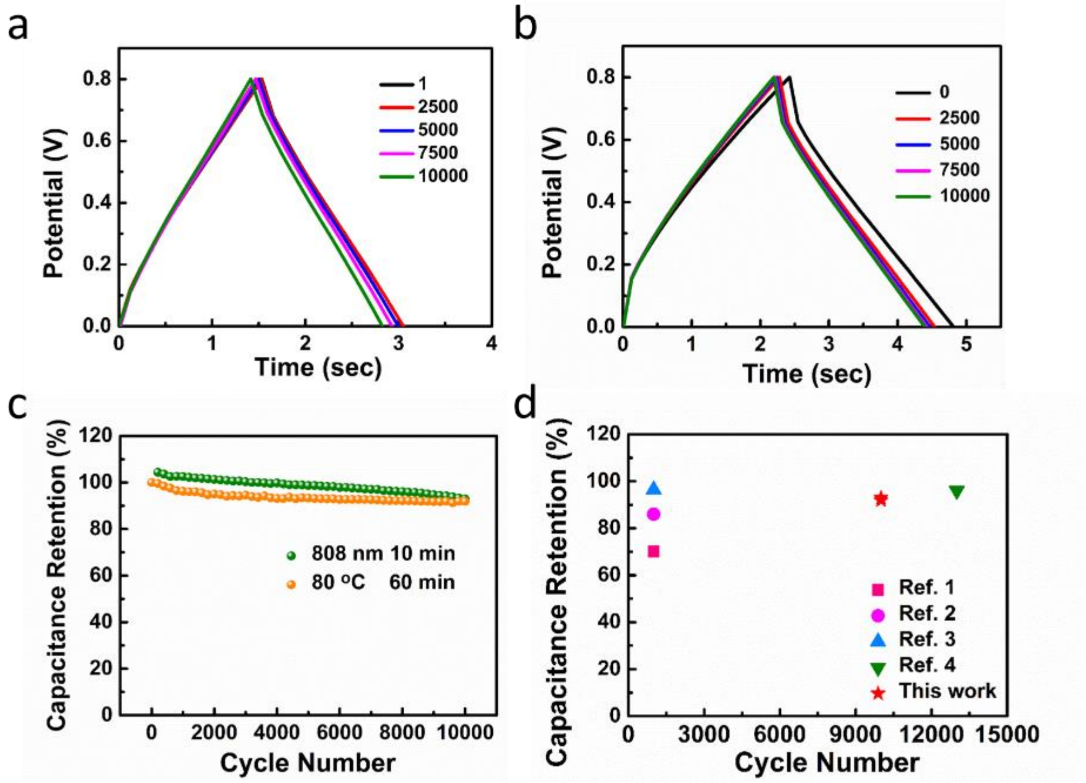

Supplementary Figure 22. GCD curves of a healed supercapacitor by 808 nm infrared light irradiation for 10 min at 1.2 mA/cm^2 (**a**), 80 ^{o}C for 60 min at 0.75 mA/cm^2 (**b**) and capacitance retentions (**c**) of healed supercapacitor performed for 10000 charge/discharge cycles. (**d**) Capacitance retentions of healed supercapacitor compared with that of as-prepared healable devices[1, 2, 3, 4].

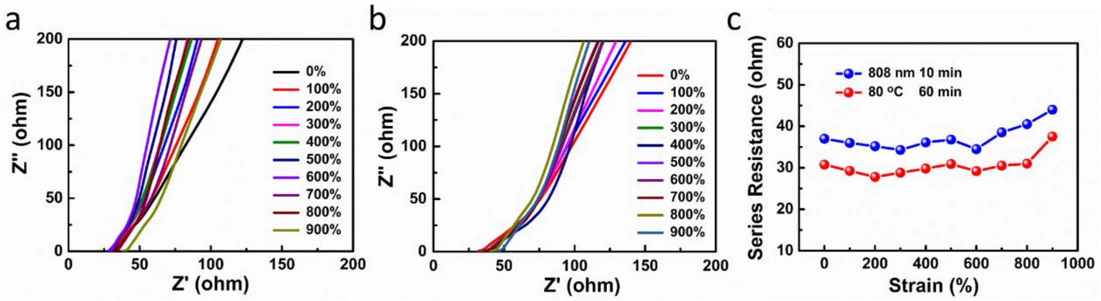

Supplementary Figure 23. EIS curves of a broken supercapacitor healed by infrared light for 10 min (**a**) and heating treatment at 80 ^{o}C for 60 min (**b**) as they were stretched from 0% to 900%. (**c**) Series resistance changes of the healed supercapacitors being stretched from 0% to 900% strain.

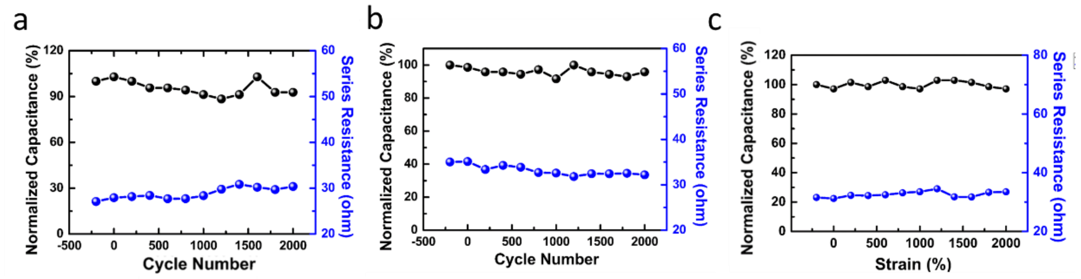

Supplementary Figure 24. Changes of specific capacitance and series resistance for a healed supercapacitor (under 80 °C for 60 min) being stretched to strains of 100% (**a**), 200% (**b**) and 300% (**c**) for 2000 cycles.

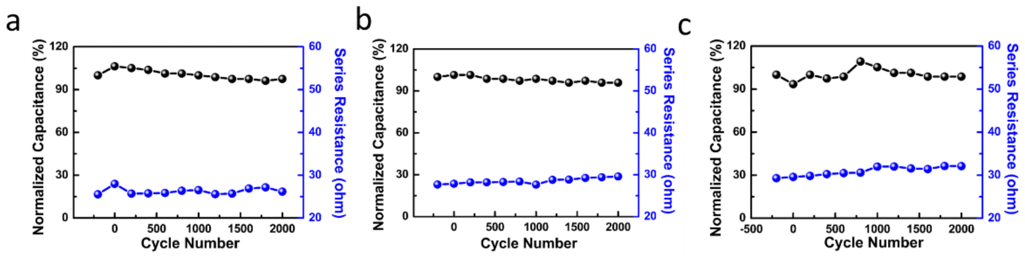

Supplementary Figure 25. Changes of specific capacitance and series resistance for a healed supercapacitor (under 808 nm infrared light irradiation for 10 min) being stretched to strains of 100% (**a**), 200% (**b**) and 300% (**c**) for 2000 cycles.

Supplementary References

1. Huang Y, *et al.* Magnetic-assisted, self-healable, yarn-based supercapacitor. *ACS Nano* **9**, 6242-6251 (2015).
2. Sun H, *et al.* Self-healable electrically conducting wires for wearable microelectronics. *Angew. Chem. Int. Ed.* **53**, 9526-9531 (2014).
3. Wang H, *et al.* A mechanically and electrically self-healing supercapacitor. *Adv. Mater.* **26**, 3638-3643 (2014).
4. Liu F, Wang J, Pan Q. An all-in-one self-healable capacitor with superior performance. *J. Mater. Chem. A* **6**, 2500-2506 (2018).
